# *In vivo* prediction of intervertebral disc strains and segmental kinematics from clinical MRI during lumbar extension

**DOI:** 10.3389/fbioe.2025.1730260

**Published:** 2026-01-12

**Authors:** Kay Ann Raftery, Saman Tavana, Becky Davis, Benjamin Thomas, Justin Lee, Julian Leong, Brett Arthur Freedman, Nicolas Newell

**Affiliations:** 1 Department of Bioengineering, Imperial College London, London, United Kingdom; 2 Fortius Clinic, London, United Kingdom; 3 Royal National Orthopaedic Hospital, Stanmore, United Kingdom; 4 UCL Institute of Orthopaedics and Musculoskeletal Science, London, United Kingdom; 5 Department of Orthopaedic Surgery, Mayo Clinic, Rochester, NY, United States

**Keywords:** digital volume correlation (DVC), disc degeneration, extension, low back pain, lumbar spine, MRI, strain, vertebra

## Abstract

**Introduction:**

Excessive intervertebral disc (IVD) strains and vertebral body motions are associated with lower back pain (LBP). Quantifying these strains and motions may aid in predicting the success of candidate LBP treatments and enable better prediction of pre-operative instability and post-operative implant failure, but cannot currently be obtained in routine clinical assessment. Thus, the aim of this study was to evaluate the feasibility of utilising clinical measures of spinal alignment, IVD geometry, and disc degeneration to predict *in vivo* IVD strains and vertebral translations.

**Methods:**

Fifteen participants presenting no LBP were subjected to one unloaded and one supine extension-loaded MRI scan. MRI-based digital volume correlation (DVC) was used to quantify the principal and shear strains of lumbar IVDs and anterior-posterior, cranial-caudal, and total translation of the vertebral bodies (L1-S1). IVD height, anterior-posterior IVD height ratio, segmental lordosis, lumbar lordosis, lumbar height, sacral angle, and Pfirrmann grade were evaluated using the reference MR images. Multivariate linear regression was used to predict level-wise strains and translations.

**Results:**

IVD strains and vertebral translations were successfully predicted from clinical measures of spinal alignment and disc degeneration, but only at the L4-L5 and L5-S1 levels. Specifically, greater minimum principal IVD strains and vertebral anterolisthesis were associated with a reduced anterior-posterior IVD height ratio at L4-L5 (p < 0.01). Greater peak minimum principal strains and anterolisthesis were associated with taller IVDs in the L5-S1 segment (p < 0.05). In the same segment, increased sacral angle was associated with greater peak minimum principal strains (p < 0.05) but lower anterolisthesis (p < 0.01).

**Discussion:**

This study demonstrates the potential of utilising radiographic variables to predict the biomechanical behaviour at the segmental level, giving rise to future exploration of complex loading patterns in patient cohorts with specific spinal pathologies.

## 1 Introduction

Up to 84% of the adult population may experience lower back pain (LBP) at some point in their lifetime (Walker, 2000), which has resulted in an estimated aggregate healthcare cost of £3.2 billion to the United States alone (Zemedikun et al., 2024). Whilst a multitude of conservative (analgesia, physical therapy, bracing) and surgical (fusion, decompression) treatment options exist, barriers to the management of LBP arise from the multi-faceted nature of LBP pathogenesis and the difficulty in mechanistic diagnoses (Allegri et al., 2016).

Excessive tissue strains of the intervertebral discs (IVDs) and motion of the vertebral bodies are thought to hallmark LBP associated with disc degeneration (Murata et al., 1994), stenosis (Lee et al., 2020), or spondylolisthesis (Dickey et al., 2002; Panjabi, 2003). Spinal instability is defined as abnormal intersegmental motion in response to physiological loading (Pope and Panjabi, 1985), disrupting the ability to protect neurological structures from irritation. For example, excessive pre-operative intersegmental motion has been hypothesised to contribute to pseudarthrosis (Heggeness et al., 1993) and other post-operative complications such as recurrent stenosis (Johnsson et al., 1989), or IVD herniation after decompression surgery (Takenaka et al., 2016).

It is currently challenging to predict which patients would benefit most from conservative therapy, and if indicated for surgical management, whether the provision of stabilisation alongside decompression is required (Simmonds et al., 2015; Dang et al., 2020). This may be due to the knowledge that instability is traditionally assessed using standing radiographs in flexion-extension positions (Dupuis et al., 1985; Murata et al., 1994; Pitkänen et al., 2002; Iguchi et al., 2004). Whilst this technique has contributed to initial knowledge of changes to spinal anatomy and instability (Iguchi et al., 2004; Leone et al., 2007), the movement can be limited by patient compliance due to pain generated by the movement, and measurements are restricted to planar translations.

Therefore, the ability to characterise 3-dimensional spinal motion would enable clinicians to make informed decisions regarding the degree of intervention needed. Supine MRI is routinely acquired as part of pre-operative evaluation or assessment of LBP, enabling surgeons or practitioners to readily obtain metrics such as Pfirrmann grade (Pfirrmann et al., 2001), IVD height, and sagittal alignment. Despite this, such images can only offer a static snapshot of patient spinal anatomy, with little knowledge surrounding the biomechanical implications of anatomical variance across patients. In parallel, the quantification of *in vivo* IVD strains and 3D vertebral body translations using MRI under supine extension has recently been validated using digital volume correlation (DVC) (Tavana et al., 2023). Yet this information remains challenging and time-consuming to acquire in a clinical environment. Therefore, it is of clinical interest to be able to predict vertebral motion and IVD deformation from conventional pre-operative metrics, especially in movements such as spinal extension, of which is routinely used for various physiotherapy exercises (Erhard et al., 1994; Mann et al., 2023; Park et al., 2024) to theoretically reduce stress in the posterior annulus (Edmondston et al., 2000).

Prior to establishing such a relationship, it is first necessary to understand the baseline interaction between lumbar vertebral translations, IVD strains, and clinically-acquired parameters, i.e., identifying the association and its directionality within a healthy cohort (presenting no LBP). The identification of variables that are associated with spinal motion and IVD deformation in a pain-free population may form a point of reference for future studies investigating cohorts with spinal pathology and presenting LBP, and may contribute towards defining a mechanism whereby spinal morphology and instability are related.

Therefore, the aim of this study was to determine whether IVD strains and segmental kinematics during extension loading could be predicted with conventional clinical measures of spinal geometry and IVD morphology in a healthy cohort.

## 2 Methods

### 2.1 Study cohort

Fifteen participants were recruited for the purposes of this study (9x male, 6x female; mean age: 37, range: 23–67) (Table 1). Participants were excluded if they reported LBP, prior spinal surgery, or pregnancy. Ethical approval was obtained from the Imperial College Research Ethics Committee (ICREC reference: 21IC6847), and written informed consent was obtained from all participants in accordance with the Declaration of Helsinki.

**TABLE 1:** Participant demographic and anthropometric details.

Participant	Gender	Age	Height (cm)	Weight (kg)	BMI
1	M	28	179	82	25.6
2	M	34	189	86	24.1
3	F	27	173	63	21.1
4	F	23	160	52	20.3
5	M	24	180	72	22.2
6	F	27	181	74	22.6
7	M	40	173	82	27.4
8	F	67	167	70	25.1
9	F	36	160	52	20.3
10	M	67	193	78	20.9
11	F	26	155	60	25.0
12	M	42	180	75	23.2
13	M	24	192	101	27.4
14	M	35	179	80	24.8
15	M	59	170	80	27.7
Mean (SD)	-	**37.3 (15.3)**	**175.4 (11.6)**	**73.8 (13.1)**	**23.8 (2.6)**

Bold typeface indicates mean (standard deviation) values.

### 2.2 Loading and MR imaging protocol

All participants were scanned twice with the chosen MR imaging sequence. Further details of the chosen MRI sequence and loading protocol can be found in (Tavana et al., 2023). Briefly, a T2 turbo spin echo sequence (voxel size: 0.58 × 0.58 × 2 mm, repetition time: 5,570 ms, echo time: 89 ms, echo train length: 17, flip angle: 148°, scan time: 14 m 36 s) was conducted on a 3T scanner (Magnetom Spectra, Siemens Medical Solution, Erlangen, Germany). This sequence was chosen from four candidate sequences as it was seen to minimise DVC errors in IVD strain and vertebral displacements (Tavana et al., 2023).

**FIGURE 1:**
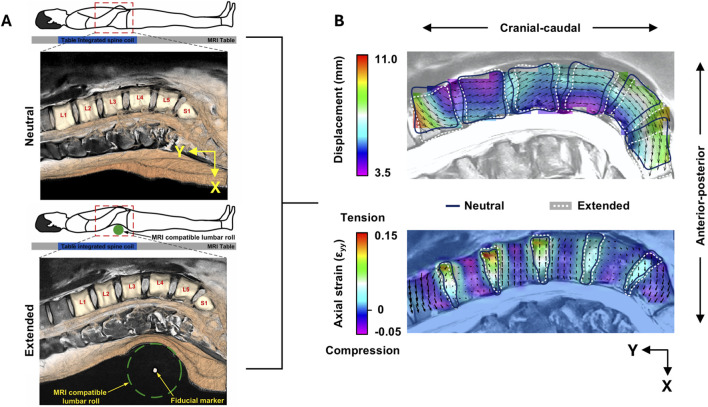
**(A)** Protocol for MRI acquisition in the neutral (top) and extension (bottom) positions. Figure adapted from Tavana et al. (2023), originally published by Elsevier under terms of the Creative Commons Attribution License (CC BY 4.0). **(B)** Mid-sagittal view of the full-field total displacement (top) and axial strain (bottom) maps for one participant. Note that the displacement and strain fields are superimposed onto the neutral position (blue mask outlines), which map onto the extended position (white dotted mask outlines and image).

In the first scan (neutral lordosis), the subject was positioned in the neutral supine position (Figure 1A). In the second scan (maximum lordosis), an MRI-compatible lumbar roll was placed in line with the L4 vertebra, verified by tracking the fiducial markers at the central axis of the roll in a rapid localiser pre-scan sequence. Participants were asked to extend their spine just under the threshold of discomfort, at which point the lumbar roll was adjusted in size to maintain this position. The use of a lumbar roll produces physiological IVD deformations and range of motion under extension (Edmondston et al., 2000) and, due to this, is indicated for patients presenting centralised LBP as part of the Mechanical Diagnosis and Therapy (MDT) exercise program (Mann et al., 2023). Furthermore, the lumbar roll has been implemented in previous MR-based studies (Beattie et al., 1994; Edmondston et al., 2000; Parent et al., 2006) to mitigate the impracticalities of actively extending the spine throughout the MRI acquisition, within the spatial restraints of the bore. This protocol has been recently validated to produce consistent kinematic outputs for each lumbar vertebrae, over six participants (Tavana et al., 2023).

### 2.3 DVC analysis

All images in the neutral and maximum lordosis positions were pre-processed according to the protocol described in (Tavana et al., 2023). Briefly, images were first cropped to exclude the posterior musculature from the field of view. Next, image voxels were made isotropic in ImageJ (v.1.53c, Bethesda, MD, US) by performing bicubic interpolation between adjacent slices (2 mm^3^–0.58 mm^3^). A binary volumetric mask was created for each of the five lumbar IVDs (L1-L2 to L5-S1) and six vertebral bodies (L1 to S1) in a commercial software (Materialise Mimics v.24, HQ, Leuven, Belgium). This was achieved using algorithmic thresholding and dilation of the region of interest (ROI) in ImageJ. Masks were then manually refined and reviewed against the raw image data to evaluate accuracy. Endplate voxels were included in the IVD masks.

DVC was conducted in DaVis (v.8.4, LaVision, Goettingen, Germany) using a Fast Fourier Transform (FFT) + Direct Correlation (DC) approach. A predictor-corrector scheme was used to achieve a final subset size of 16 voxels (4.64 mm) (48–32-24–16, passes: 1-1-2-2, subset overlap: 50%, minimum valid voxel: 50%), where the subset size was chosen based on recommendations from a prior investigation of MRI-based *in vivo* DVC (Tavana et al., 2023).

To account for small movements between successive MR scans and increase the efficiency of the DVC algorithm, rigid body translations and rotations were removed prior to performing the DVC analysis (Mostafavi et al., 2015). This was performed using the in-built function in DaVis, which determines the common rigid body shift of the total volume and rigid body rotations about the centroid through polar decomposition. This transformation is then removed from the calculated displacement field. Given the expected high deformation of posterior musculature tissue during extension, cropping the images during pre-processing to remove this tissue meant that rigid body movement calculations were not susceptible to artefacts from the peripheral anatomy.

The average of the displacement fields within the vertebral body masks were used to calculate the translations in the x (Vx: anterior-posterior, positive = posterior), y (Vy: cranial-caudal, positive = cranial), and z (Vz: lateral, positive = right) directions (Figure 1B). Total translation (Vtot) was defined as the absolute 3D translation calculated from the resolved components. This DVC protocol has been shown to generate a vertebral displacement precision of 0.165 mm (Vx), 0.129 mm (Vy), 0.138 mm (Vz), and 0.251 mm (Vtot), and an IVD strain accuracy and precision of 0.34% and 0.18%, respectively (Tavana et al., 2023).

To account for relative anterior-posterior translations between superior and inferior vertebra within each segment, the anterolisthesis (anterior translation of the superior vertebra relative to the inferior) was calculated. This was defined as the difference in Vx between the superior and inferior vertebra, where a greater positive number indicates greater anterolisthesis, and a greater negative number indicates greater retrolisthesis.

For the displacement fields within the IVD masks, a custom-written script in MATLAB (MathWorks, Inc., Natick, MA, US) was used to calculate the 3-dimensional Green-Lagrangian strain tensor from the displacements, and further calculate the maximum principal, minimum principal, and maximum shear strains (Figure 1B). Strains from each subset were averaged over the IVD volume to acquire mean strain, and peak strain was defined as the subset with the highest magnitude of strain within the region of interest.

### 2.4 Measurement of geometrical and morphological variables

The following variables were measured at each individual segment (L1-L2 to L5-S1): Pfirrmann grade, anterior, central, and posterior IVD height, anterior-posterior IVD height ratio, and segmental lordosis. At the lumbar spine level, lumbar lordosis, lumbar height, and sacral angle were measured. All geometric variables were quantified in a clinical image navigation software (Codonics Clarity Viewer, OH, US) using the mid-sagittal slice of the MRI in neutral lordosis.

**FIGURE 2:**
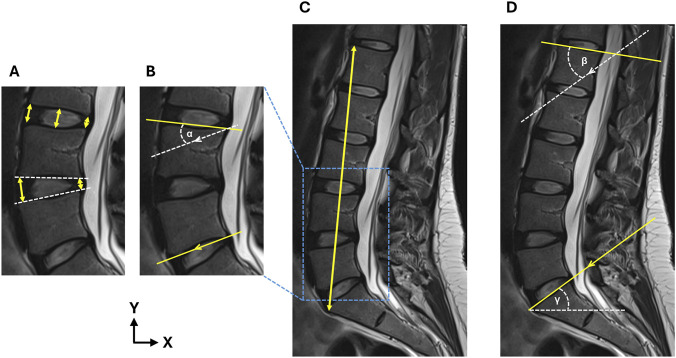
**(A)** Measurement of the anterior, central, and posterior IVD heights to calculate the anterior-posterior IVD height ratio, **(B)** measurement of the segmental lordosis angle (α), **(C)** measurement of lumbar height, and **(D)** measurement of lumbar lordosis (β) and sacral angle (γ). Arrows denote parallel line pairs.

To measure IVD height, a line was drawn between the inferior endplate of the superior vertebra and superior endplate of the inferior vertebra, which was orthogonal to the mid-IVD plane. The line was drawn at the anterior, central, and posterior of the IVD (Figure 2A) (Lin et al., 2016). The anterior-posterior IVD height ratio was calculated by dividing the anterior height by the posterior height (Figure 2A).

To measure segmental lordosis at each IVD level, the Cobb method was implemented (Schuler et al., 2004). An angle was subtended between a line drawn parallel to the superior endplate of the superior vertebral body, and a line parallel to the inferior endplate of the inferior vertebral body (Figure 2B). Where endplates displayed notable concavity, the subtended line was parallel to the plane joining the anterior and posterior extremities of the endplate rim. Negative angles were defined as kyphotic and positive angles were defined as lordotic.

Lumbar height was measured between the most anterior point of the superior L1 endplate and the S1 superior endplate (Deng et al., 2015) (Figure 2C). Total lumbar lordosis angle was measured as the angle between the superior endplate of the L1 and S1 vertebral bodies (Lord et al., 1997; Frenkel et al., 2018) (Figure 2D). Sacral slope was measured as the angle subtended by a line parallel to the superior S1 endplate relative to the horizontal (O’Brien et al., 2008; Pytiak et al., 2016) (Figure 2D).

Finally, disc degeneration for all five lumbar IVDs was quantified using the Pfirrmann scale (Pfirrmann et al., 2001) by three experienced raters (ST, KR, and NN). The Pfirrmann scale qualitatively describes the structure and signal intensity of the IVD (1: homogenous and bright; 5: inhomogeneous and black), the nucleus-annulus distinction (1: clear; 5: lost), and the IVD height (1: normal; 5: collapsed). The final Pfirrmann grade was a rounded average of all three ratings. Grades 1 and 2 were considered “non-degenerated” (ND), Grade 3 was considered “degenerated” (D), and Grades 4 and 5 were considered “severely degenerated” (SD).

### 2.5 Statistical analysis

Statistical analysis was performed in SPSS (v29, IBM corp., Armonk, NY, US). Reliability of geometrical measurements were assessed by two raters (ST and KR) over a random sample of three participants. For each participant, geometrical measures were independently performed by both raters for all lumbar levels. The inter-rater intraclass correlation coefficient (ICC) was calculated using a two-way random effects model for absolute agreement (Koo and Li, 2016).

Backwards multivariate linear regression was used to predict IVD mean strains, peak strains, and vertebral translations (Vx, Vy, Vz, and Vtot) for each vertebral level. All geometric variables, Pfirrmann grade, and participant age, gender, and BMI were input as model covariates. Multicollinearity was assessed by inspection of variation inflation factors (VIF), where a VIF <10 was considered acceptable. Covariates were removed from the initial model where the VIF was above this threshold. Additionally, in all models, normality was verified by plotting the observed cumulative probability against the observed, and homoscedasticity was verified by plotting the Pearson residuals against the predicted values. Models which violated these assumptions were not analysed further.

Since two vertebral bodies were associated with one lumbar segment (six vertebral body translations *versus* five lumbar IVDs and segments), the models with vertebral translation as the independent variable were repeated twice. In the first model, the IVD was matched with the adjacent superior vertebral level, e.g., the L1 vertebra translation was predicted by the measurements of the L1-L2 segment. In the second model, the IVD was matched with the adjacent inferior vertebral level.

Hommel correction was used to correct the Family-Wise Error Rate within each group of tests performed per level. Alpha was set to 0.05.

## 3 Results

### 3.1 Reliability of clinical measurements

The ICC of all geometrical measures were deemed acceptable for further analysis (Table 2), where reliability between raters was considered “excellent” (ICC >0.9) apart from posterior IVD height, which was considered “good” (ICC >0.8) (Koo and Li, 2016).

**TABLE 2:** Intra-class correlation coefficients and 95% confidence intervals for all geometrical variables.

Variable	ICC^a^ (95%CI)
Anterior disc height	0.817 (0.548–0.934)***
Central disc height	0.959 (0.883–0.986)***
Posterior disc height	0.847 (0.584–0.947)***
Segmental lordosis	0.984 (0.947–0.995)***
Total lordosis	0.992 (0.513–1.000)***
Lumbar height	0.983 (0.331–1.000)**
Sacral angle	0.957 (0.391–0.999)*

***p < 0.001, **p < 0.01, *p < 0.05.

^a^ Intra-class correlation coefficient.

### 3.2 Cohort description of IVD strains and vertebral displacements

**FIGURE 3:**
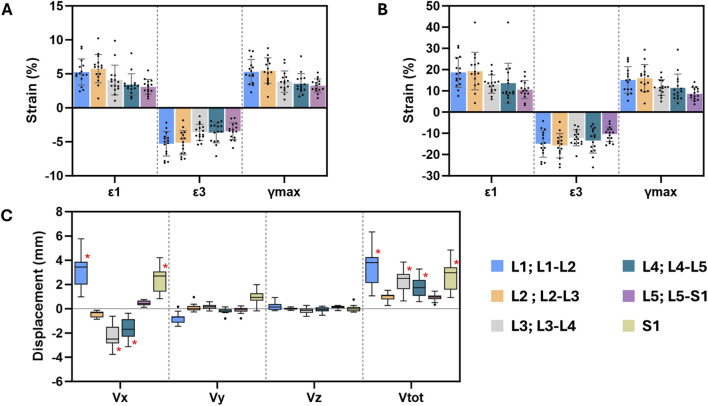
Mean (SD) of **(A)** mean and **(B)** peak maximum principal (ε1), minimum principal (ε3), and maximum shear (γmax) strains for each lumbar IVD. **(C)** Median and interquartile ranges of vertebral displacements of all lumbar levels. Red asterisk highlights the measurements which suitably surpass the respective precision estimate.

Mean and peak IVD strains, and mean vertebral displacements, are depicted in Figure 3 for all lumbar levels. IVD deformation magnitudes were comparable across levels (Figures 3A,B). In contrast, displacements were largely dependent on the vertebral level (Figure 3C). The largest source of kinematic variation was at L1, where Vtot ranged from 1.060 to 6.340 mm. In some cases, displacements were indistinguishable from the error margin, where mean values or the range of values were less than an order of magnitude higher than the respective precision estimate (Figure 3C) (Dall’Ara et al., 2017). Regression models pertaining to these raw quantities were not analysed further.

### 3.3 Prediction of mean and peak IVD strains

Due to multicollinearity (VIF >10), anterior IVD height, central IVD height, posterior IVD height, and total lumbar lordosis angle were removed as predictor variables in all models, and age was additionally removed as a predictor in L1-L2 models. BMI was additionally removed as a predictor in L4-L5 models, and produced no significant univariate correlations with any target variable at this level (*p* > 0.334).

**FIGURE 4:**
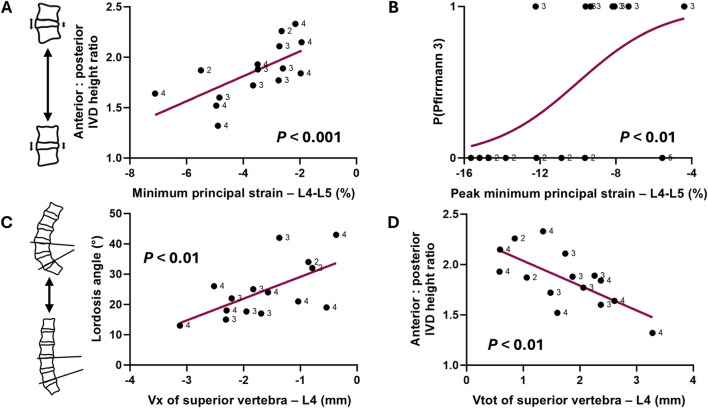
Relationships at L4-L5 between **(A)** minimum principal IVD strain (negative = compressive strain) and anterior: posterior IVD height ratio, **(B)** peak minimum principal IVD strain and probability of presenting moderate degeneration, or a Pfirrmann 3 IVD (P(Pfirrmann 3)), **(C)** anterior-posterior L4 translation (Vx, negative = anterior) and lordosis angle, and **(D)** total L4 translation (Vtot) and anterior: posterior IVD height ratio. Note that each datapoint is labelled with Pfirrmann grade.

Multivariate models to predict mean IVD strains were significant only in the L4-L5 and L5-S1 segments (Table 3). In the L4-L5 segment, greater minimum principal strains (more negative strains) were associated with lower anterior-posterior IVD ratio (β = 5.23, *p* < 0.001) (Figure 4A), older age (β = −0.47, *p* < 0.05), and non-degenerated IVDs (β = −2.39, *p* < 0.05).

**TABLE 3:** Significant multivariate linear regression models for the prediction of IVD strains. Dashed boxes indicate that the parameter did not reach statistical significance within the model.

Model parameter	L4-L5 minimum principal strain			L5-S1 peak minimum principal strain		
F	9.98			8.64		
Adjusted *R* ^2^	0.66			0.77		
Corrected p	0.022			0.044		
Predictor	B	SE^a^	p	B	SE	p
Age	−0.47	0.18	0.022	−0.17	0.06	0.022
Gender (male)	-	-	-	5.85	1.47	0.004
Average IVD height	-	-	-	−1.91	0.37	<0.001
Anterior: posterior IVD height ratio	5.23	0.97	<0.001	-	-	-
ND^b^ (Pfirrmann 2)	−2.39	0.78	0.011	-	-	-
MD^c^ (Pfirrmann 3)	-	-	-	5.15	1.16	0.002
SD^d^ (Pfirrmann 4 & 5)	-	-	-	​	​	​
Segmental lordosis	-	-	-	0.29	0.09	0.011
Lumbar height	-	-	-	​	​	​
Sacral angle	-	-	-	−0.18	0.059	0.015
Constant	−11.27	1.64	<0.001	4.84	4.64	0.33

^a^ Standard error.

^b^ Non-degenerated.

^c^ Moderately degenerated.

^d^ Severely degenerated.

In the L5-S1 segment, peak minimum principal strains were associated with greater IVD height (β = −1.91, *p* < 0.001), reduced segmental lordosis angle (β = 0.29, *p* < 0.05), but an increased sacral angle (β = −0.18, *p* < 0.05). Additionally, older, male participants tended to exhibit higher peak minimum principal IVD strains, whilst moderately degenerated IVDs were associated with lower strains (Table 3; Figure 4B).

### 3.4 Prediction of vertebral body translations

**FIGURE 5:**
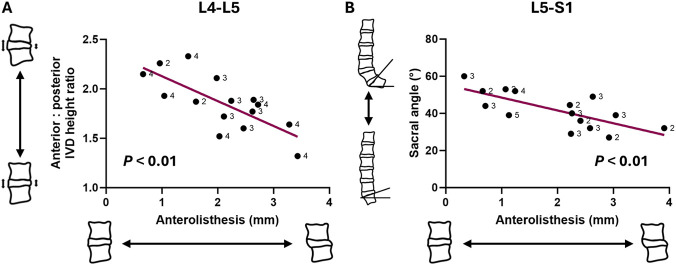
Correlation plots between spinal metrics and anterolisthesis in the **(A)** L4-L5 segment and **(B)** L5-S1 segment. Note that each datapoint is labelled with Pfirrmann grade.

Models to predict vertebral body translations were significant in the L4-L5 and L5-S1 segments (all *p* < 0.05) (Table 4). In the L4-L5 segment, greater anterior translation (Vx) of the L4 vertebra was associated with a reduced lordosis angle (β = 0.04, *p* < 0.01) (Figure 4C) and lower anterior-posterior IVD height ratio (β = 1.74, *p* < 0.01). The same association was observed between these two variables and overall movement of the L4 vertebra (Vtot) (Table 4) (Figure 4D). Additionally, the degree of anterolisthesis in the L4-L5 segment was negatively associated with the anterior-posterior IVD height ratio (β = −2.02, *p* < 0.01) (Table 4) (Figure 5A).

**TABLE 4:** Significant multivariate linear regression model outputs for the prediction of vertebral body translations. Dashed boxes indicate that the variable did not reach statistical significance within the model. Variables with no significant effect in any model are not shown.

Model parameter	L4-L5								L5-S1						
	Vx of superior vertebra (L4)			Vtot of superior vertebra (L4)					Anterolisthesis				Anterolisthesis		
F	16.77			15.56					11.41				15.22		
Adjusted *R* ^2^	0.69			0.68					0.66				0.67		
Corrected p	0.013			0.013					0.022				0.015		
Predictor	B	SE	p	B		SE	p		B		SE	p	B	SE	p
Gender (male)	-	-	-	-		-	-		-		-	-	-	-	-
Average IVD height	-	-	-	-		-	-		-		-	-	0.15	0.05	0.016
Anterior: Posterior IVD height ratio	1.74	0.43	0.002	−1.69		0.43	0.002		−2.02		0.51	0.002	-	-	-
MD^a^ (pfirrmann 3)	-	-	-	-		-	-		-		-	-	-	-	-
Segmental lordosis	0.04	0.01	0.005	−0.04		0.01	0.007		−0.03		0.02	0.08	-	-	-
Sacral angle	-	-	-	-		-	-		-		-	-	−0.07	0.02	0.002
Constant	−5.95	0.82	<0.001	5.91		0.82	<0.001		6.549		0.96	<0.001	3.28	0.97	0.006

^a^ Moderate disc degeneration.

In the L5-S1 segment, no individual vertebral translation components were associated with any of the measured variables. However, anterolisthesis was predicted by a greater average IVD height (β = 0.15, *p* < 0.05) and reduced sacral angle (β = −0.07, *p* < 0.01) (Table 4) (Figure 5B).

## 4 Discussion

The present study demonstrates the existence of relationships between 3-dimensional internal IVD strains, segmental kinematics, and anatomical measures that can be made from standard clinical images within a cohort presenting no LBP. Understanding how sagittal alignment and IVD morphology affect spinal motion in such a population is necessary, as the parameters flagged in this study (e.g., segmental lordosis, sacral angle) may be indicative of instability outside of the healthy range reported here, thus could be used to optimise the choices of covariates when investigating a pathological population.

IVD strains and notable motion patterns were only associated with clinically measured variables at the L4-L5 and L5-S1 segment. This finding bears clinical relevance, as the L4-L5 and L5-S1 segments are the most common to require surgical intervention, with the highest incidence of pathological events such as herniation (Jordan et al., 2009), spondylolisthesis (Hey et al., 2024), and stenosis (Tomkins-Lane et al., 2014). Prior studies have highlighted the L5-S1 segment for its tendency to produce anterolisthesis in functional myelography, whilst other segments produce negligible motion (Johnsson et al., 1989). However, this finding may in part be related to the resolution constraints of the DVC, where higher lumbar levels generated motion that did not surpass the error threshold to a sufficient degree.

In L4-L5 and L5-S1, the presence of non-degenerated (Pfirrmann 2) IVDs were significantly associated with the increase in mean and peak minimum principal IVD strains during extension (Table 3). Disc degeneration has been shown to be associated with anterior migration of the nucleus in response to extension moments (Tsantrizos et al., 2005), which could alleviate posterior compressive stresses. Additionally, O’Connell et al. (2011) observed that in extension, non-degenerated IVDs were more likely to exhibit higher radial strains in the posterior annulus than degenerated IVDs using high-resolution MRI (O’Connell et al., 2011); this finding at the apparent scale could manifest as greater compression at the whole-IVD level observed in the present study, where individual lamellae cannot be resolved.

In the L4-L5 segment, lower anterior-posterior IVD height ratio and reduced segmental lordosis were associated with higher minimum principal IVD strains (Figure 4A). This suggests that those with reduced lordosis, or “flat back syndrome” (Lu and Chou, 2007), exhibit high compressive strains during extension, likely in the posterior annulus. Given that maximum principal strains – likely prominent in the anterior annulus – were not also significantly increased when angulation of the segment decreased, it could suggest a more anterior pivot point in the IVDs of those with straighter spines as a compensatory response to extension loading. It is hypothesised that some patients presenting LBP with reduced segmental lordosis do not exhibit this compensation response, and as a result may be less suited to extension-based physiotherapy. In some instances, a lumbar roll may be prescribed in supine or sitting movements (Erhard et al., 1994; Takasaki et al., 2018). Therefore, lumbar kinematic data upon the application of such equipment could be used as a benchmark in future investigations into patient suitability of extension-based physiotherapy.

Previous work has demonstrated that the variation in IVD strains and vertebral translations between individuals is significantly smaller than the variation between lumbar levels (Tavana et al., 2023), suggesting that a “healthy” range of strain and translation exists for individuals reporting no LBP. Spinal alignment metrics during extension loading have become important in predicting the risk of post-operative complications (Takenaka et al., 2016). In the present study, lordosis angle and anterior-posterior IVD height ratio was negatively associated with greater anterolisthesis at the L4-L5 segment (Figure 5) – in other words, straighter segments indicated greater forward “slippage” of the L4 vertebra. However, it has been shown that reduced lordotic angle paired with increased retrolisthesis of the superior vertebra significantly increased the risk of herniation after decompression surgery (Takenaka et al., 2016). The present study, taken together with Takenaka et al. (2016) findings, demonstrates that particular combinations of geometrical measures and identifiable movement patterns may be characteristic of the development of spinal pathology in certain cohorts. Thus, further work is needed to characterise the 3-dimensional internal IVD strains and vertebral kinematics during extension loading in non-healthy cohorts.

Clinically, anterolisthesis or retrolisthesis of one vertebra relative to another, accompanied by the loss of lumbar lordosis, is characteristic of degeneration-related LBP (Barrey et al., 2007). However, it is unclear as to whether lumbar lordosis or segmental angle contributes to the development of pathological events such as spondylolisthesis (Berlemann et al., 1999; Chen and Wei, 2009; Abu-Leil et al., 2016). For instance, unlike the present study, Lee et al. (2021) found no relation between IVD angle and instability in patients with spondylolisthesis (Lee et al., 2021). This highlights that relationships between clinical parameters and vertebral motion may be distinct in pathological *versus* control cohorts, although the use of 2-dimensional measures and the diagnosis of instability through flexion motions in Lee et al. (2021) may in part explain the discrepancy. Future work capturing MRIs in the flexed position is required for a comprehensive evaluation of the how spinal geometry can influence lumbar kinematics.

The inability to capture motion from extension to flexion may explain the somewhat surprising finding that disc degeneration was not associated with lumbar anterolisthesis. It is likely that the extension alone is insufficient to capture this motion (Boden and Wiesel, 1990), and furthermore, due to restabilisation of the joint as degeneration progresses (Yong Hing and Kirkaldy Willis, 1983), any subluxation observed on static radiographs may not correspond to the motion observed dynamically (Boden and Wiesel, 1990).

Sacral angle displayed significant relationships with both IVD minimum principal strain and anterolisthesis of the L5-S1 segment only (Tables 3, 4). In particular, a greater sacral angle was associated with higher compressive IVD strains, which is counterintuitive to reduced segmental lordosis angle being a parallel predictor. However, the relationship between sacral angle and lumbar lordosis is not strictly positive nor linear, and largely determined by the lordosis inflection point and apex (Roussouly et al., 2005). Based on this, participants in this study with high compressive strains at the L5-S1 IVD are more likely to exhibit “type 4” lordosis, defined by a more cranial lordosis apex (e.g., at the L3 vertebra) (Roussouly et al., 2005). It is interesting to note that spinal stenosis is linked to “type 4” lordosis (Roussouly et al., 2005). It could be hypothesised that flexion-extension movements with this posture could generate excessive IVD strains in the lower lumbar region, which over time could predispose the IVD to annular delamination and outwards bulging (Tavana et al., 2021).

A limitation of this study is that only 15 healthy participants were recruited, which means caution should be applied when interpreting findings in relation to spinal pathologies and clinical outcomes. However, as a proof-of-concept study, the small sample was sufficient to understand whether such correlations could be drawn between conventional clinical measures and biomechanical quantities, potentially landmarking a shift in the way pre-operative metrics are utilised and interpreted. Thus, future work which focusses on cohort expansion and the stratification of patients undergoing intervention for LBP-related pathologies is warranted to eventually lead to improved clinical outcomes.

Additionally, supine positioning causes altered sagittal alignment compared to standing (Tian et al., 2025). These associations are therefore only applicable for supine geometrical measures. However, it been demonstrated that anterior sagittal translation is significantly greater in weight-bearing standing radiographs relative to supine (Lowe et al., 1976), suggesting that effect sizes may be under-estimated in this study. Another limitation was that translation components were resolved relative to the global axis, which meant that it was not possible to report anterior-posterior and cranial-caudal translations respective to the orientation of the vertebral body. Again, vertebral translations may have therefore been under-estimated with the local axes orthogonal to the global co-ordinate system. However, the separation of regression models into respective lumbar levels may have somewhat standardised vertebral inclination and thus minimised this confounding effect.

Lastly, in some participants, Vx displacements did not exceed 10 times that of the precision estimate (Dall’Ara et al., 2017). Particular caution should be applied to the anterolisthesis cases, where propagation of error would see the precision estimate to be 0.330 mm, and that meaningful displacements were not observed at the L5 vertebra (Figure 3C). However, the translational value of having access to physiological measurements of internal kinematics may supersede error criteria which are typically based on *in vitro* data. Human *in vivo* imaging protocols are inherently limited in their ability to produce errors on a similar scale (an issue also faced by Oravec et al. (2025)), and thus, the spatial resolution of the displacement field should be compromised accordingly, but not so much that the subset size exceeds the dimensions of the tissue being studied (Dall’Ara et al., 2017). In this instance, the subset size was optimal at 16 voxels (4.64 mm) as larger subsets would not be able to discern axial strains within the IVD, where the average disc height recorded in this cohort was approximately double the subset size (9.6 mm).

## 5 Conclusion

This study demonstrates that measures of sagittal angle such as segmental lordosis and anterior: posterior IVD height ratio are positively associated with IVD compressive strains and anterior translation of the vertebra cranial to the segment. These associations were restricted to the lower lumbar levels. Results suggest that in the future, it may be possible to infer patient-specific spinal movement patterns from static radiographic measurements, which may assist in the streamlining of clinical decision making to improve quality of care in patients with LBP.

## Data Availability

The raw data supporting the conclusions of this article will be made available by the authors, without undue reservation.
